# Geographic separation and genetic differentiation of populations are not coupled with niche differentiation in threatened Kaiser’s spotted newt (*Neurergus kaiseri*)

**DOI:** 10.1038/s41598-019-41886-8

**Published:** 2019-04-17

**Authors:** Forough Goudarzi, Mahmoud-Reza Hemami, Loïs Rancilhac, Mansoureh Malekian, Sima Fakheran, Kathryn R. Elmer, Sebastian Steinfartz

**Affiliations:** 1 Department of Natural Resources, Isfahan University of Technology, Isfahan, 8415683111 Iran; 2 Department of Evolutionary Biology, Unit Molecular Ecology, Zoological Institute, Technische Universität Braunschweig, 38106 Braunschweig, Germany; 3 Institute of Biodiversity, Animal Health & Comparative Medicine, College of Medical, Veterinary & Life Sciences, University of Glasgow, Glasgow, G12 8QQ UK; 4 University of Leipzig, Institute of Biology, Molecular Evolution and Systematics of Animals, Talstrasse 33, 04103 Leipzig, Germany

**Keywords:** Conservation biology, Ecological genetics, Ecological modelling

## Abstract

The combination of niche modelling and landscape genetics (genomics) helps to disentangle processes that have shaped population structure in the evolutionary past and presence of species. Herein, we integrate a comprehensive genomic dataset with ecological parameters and niche modelling for the threatened Kaiser’s newt, a newt species adapted to mountain spring-ponds in Iran. Genomic analysis suggests the existence of two highly differentiated clades North and South of the Dez River. Genetic variation between the two clades (76.62%) was much greater than within clades (16.25%), suggesting that the Dez River prevented gene flow. River disconnectivity, followed by geographic distance, contributed mostly to genetic differentiation between populations. Environmental niche and landscape resistance had no significant influence. Though a significant difference between climatic niches occupied by each clade at the landscape-scale, habitat niches at the local-scale were equivalent. ‘Niche similarity analysis’ supported niche conservatism between the two clades despite the southward shift in the climatic niche of the Southern clade. Accordingly, populations of different clades may occupy different climatic niches within their ancestral niche. Our results indicate that the change of climatic conditions of geographically and genetically separated populations does not necessarily result in the shift of an ecological niche.

## Introduction

Landscape characteristics influence functional connectivity of habitats and therefore affect migration and gene flow of individuals among populations^1^. These characteristics can be biotic or abiotic, natural or anthropogenic, making several combinations possible, such as abiotic-anthropogenic (roads and modified topography), biotic-anthropogenic (agriculture), natural-abiotic (water resources distribution) and finally natural-biotic (community composition)^2^. Landscape factors affecting the level of genetic differentiation among populations can be summarised as ‘geographic distance’, ‘landscape resistance’ and ‘environmental resistance^3^.

Isolation by distance (IBD) is the correlation between the genetic distance of individuals or populations compared to their geographic distance^4^. As in a heterogeneous environment, gene flow may be limited such that straight-line geographic distances may actually not represent the real geographic distances underlying observed genetic distance^5^. Accordingly, species that require specific habitat conditions may not disperse or migrate directly to a new suitable habitat patch; instead, landscape characteristics may promote or resist individual migration. Amphibians are typically associated with wet or humid habitat patches that are interrupted by non-suitable habitat patches. For instance, dry lands can act as movement barriers for salamanders^6^, though gene flow sometimes occurs through matrix habitats temporarily turned into suitable habitat^7^. Therefore, functional connectivity describes the amount of genetic variation explained by landscape resistance, the pattern underlain by isolation by resistance (IBR)^8^. In addition, the balance between gene flow and selection in populations that occupy different environments determines the population genetic differentiation^9,10^. In such circumstances, adaptation to local environmental conditions will diminish the establishment success of migrants and effective gene flow will decrease. Such local adaptations lead to a pattern of isolation by environment (IBE). Isolation by environment examines the effect of environmental niche dissimilarity causing genetic differentiation^11^.

Genetic divergence at the intraspecific level is mainly the result of limited gene flow among allopatric populations or lineages. If these lineages also use different components of the environmental space, this differentiation will result in a ‘niche divergence’ pattern^12^. In contrast, closely related lineages may tend to retain their ancestral niche-related traits over time (phylogenetic niche conservatism; PNC^13^). An intermediate pattern between these two extremes would be ‘niche constraint’ (sensu Pyron, *et al*.^14^) in which two lineages segregate in environmental analogues of their ancestral niche^14^. Taking either of these evolutionary paths depends on many factors, including the degree of geographic and environmental heterogeneity, the lineage’s genetic predisposition, and timescale^14^. PNC assists in forecasting species geographic responses to changes in large scale environmental conditions that might be relevant to climate change (e.g. temperature and precipitation). Under global climate change conditions, species with conserved climatic niches might be driven to extinction if they are not able to adapt to changing conditions, e.g. by shifting their range^15^. To retain the ancestral climatic niche in a changing environment, populations may need to shift their geographical range; a task that may be difficult or impossible in heterogeneous landscapes. Especially for amphibians, which are in general characterised for limited dispersal propensity (but see^16^), lineages with strongly conserved niche may remain isolated in suitable habitat patches or are at risk of extirpation if they cannot migrate.

Members of the genus *Neurergus* are found in the Near East and form a monophyletic clade of newts within the family of Salamandridae that have adapted to a mountainous habitat reproducing primarily in streams^17–19^. Up to five species are  currently recognized^20–22^, among those the distribution of *Neurergus kaiseri* (Kaiser’s newt) extends to Iran, where which is the most southern distribution range of the genus and all Salamandridae in Eurasia. According to the IUCN red list assessment, *N*. *kaiseri* is classified as vulnerable^23^; it is restricted to springs in the southern Zagros Mountains of Iran. Across its geographical range, populations of *N*. *kaiseri* are facing a heterogeneous mountainous landscape with diverse climatic conditions ranging from wet to dry that is disconnected by two main rivers, Dez and Karoon. This is in contrast to the remaining more northerly distributed species (*N*. *strauchii*, *N*. *crocatus, N. barrani* and *N*. *derjugini*), which occupy more homogenous mountainous habitats and reproduce in streams. As a result of its southern distribution and the lack of consistent water supply of available streams, Kaiser’s newt is the only spring-pond-breeding species of the genus. In general, *N*. *kaiseri* can be characterised as a spring-pond-breeding lineage of newts that occupies suitable habitat patches in a rather heterogeneous environmental setting with limited migration between suitable patches. A first insight on the structure of *N*. *kaiseri* populations based on the mitochondrial D-loop indicated the existence of two clades, with their divergence best explained by geographic distance^24^. However, the structure inferred from a single locus such as the mtDNA can substantially differ from a multi-locus approach of genomic DNA, which is reflecting more accurately the true history of populations and at higher resolution. Thus, in order to obtain a more complete understanding of both genetic structure and the underlying processes shaping it, the analysis of an extensive multi-locus dataset for *N*. *kaiseri* is crucial to inform future conservation measures.

We sampled thousands of loci across the nuclear genome by applying Restriction Associated DNA sequencing (RADseq). This dataset was then used to perform a comprehensive population structure analysis, which is the basis to address the question in how far landscape characteristics have shaped and impacted the observed population structure. We hypothesised that geographic distance (IBD) and environmental niche dissimilarity (IBE) acting as an adaptation barrier, and that landscape resistance (IBR) and barriers such as rivers (isolation by river, IBRiv) would limit gene flow among populations of *N*. *kaiseri*. Specifically we explored (1) to what extent *N*. *kaiseri* clades kept the same climatic tolerance after separation, (2) whether niche divergence/conservatism accelerates adaptation and diversification within this species, and (3) which ecological factors cause the species niche to be conserved at coarse and fine scales^14^.

## Results

### RADseq dataset

**Figure 1:**
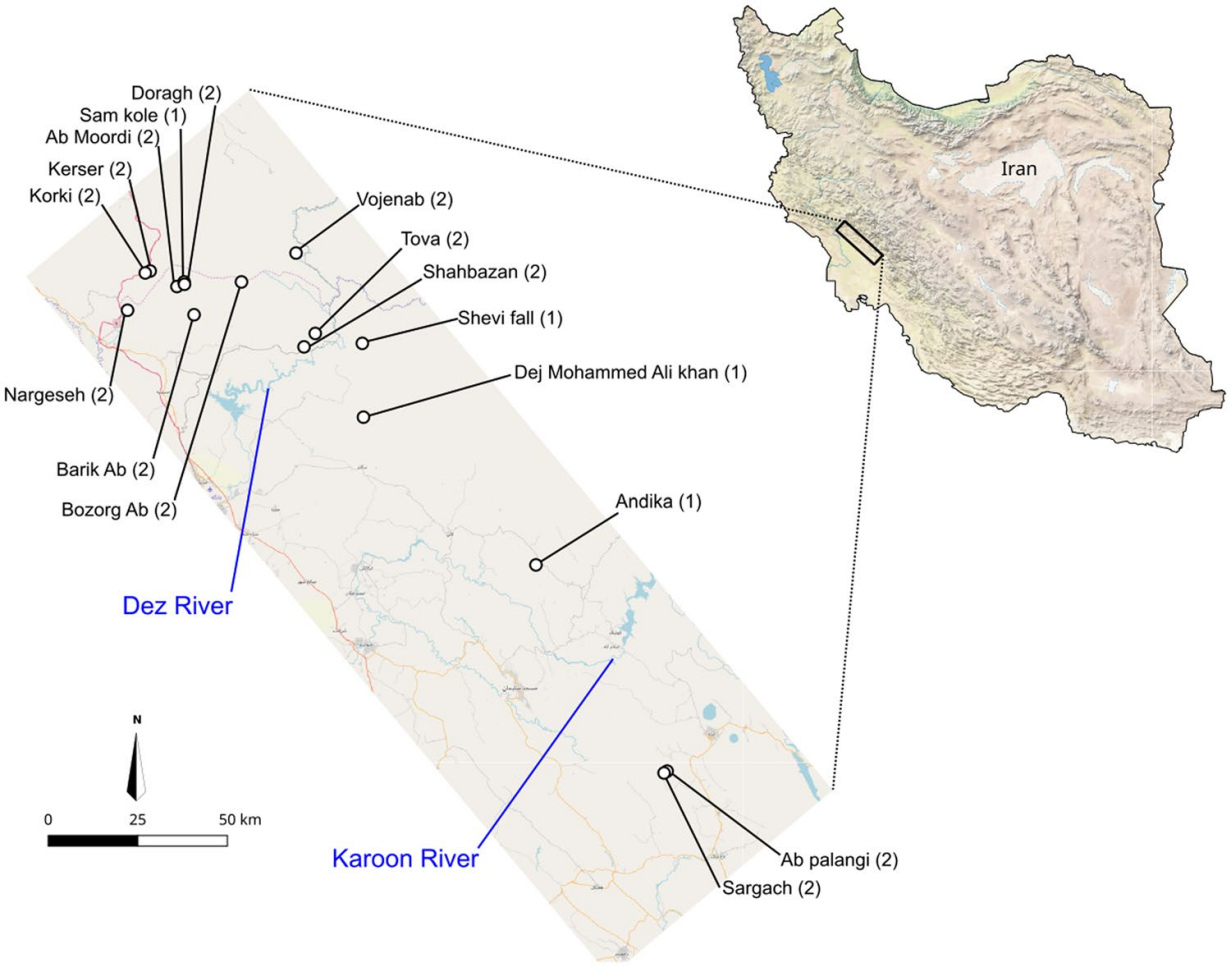
Map of the sampling localities of *Neuregus kaiseri* across the southwestern Zagros Mountains, Iran, with the sampling size indicated between parentheses. The two major rivers of the area are also indicated. The maps were generated in ArcGIS 10.4 using the base map from ^©^OpenStreetMap contributors (https://www.openstreetmap.org), available under ODbL (www.openstreetmap.org/copyright).

Using *de novo* assembly on reads obtained from a ddRAD protocol (modified from^25^), we recovered a total of 26,746 loci of an average length of 115 bp (total of 3,080,681 bp), each of them being present in at least 28 individuals of *N*. *kaiseri* from 16 localities (Fig. 1). From these loci, 18,649 SNPs were identified, and all individuals were genotyped at these sites. When including outgroups (one individual of both *N*. *crocatus* and *N*. *derjugini*) to perform phylogenetic analyses, 23,518 loci were recovered for a total of 2,709,284 bp.

### Population genetic structure

**Figure 2:**
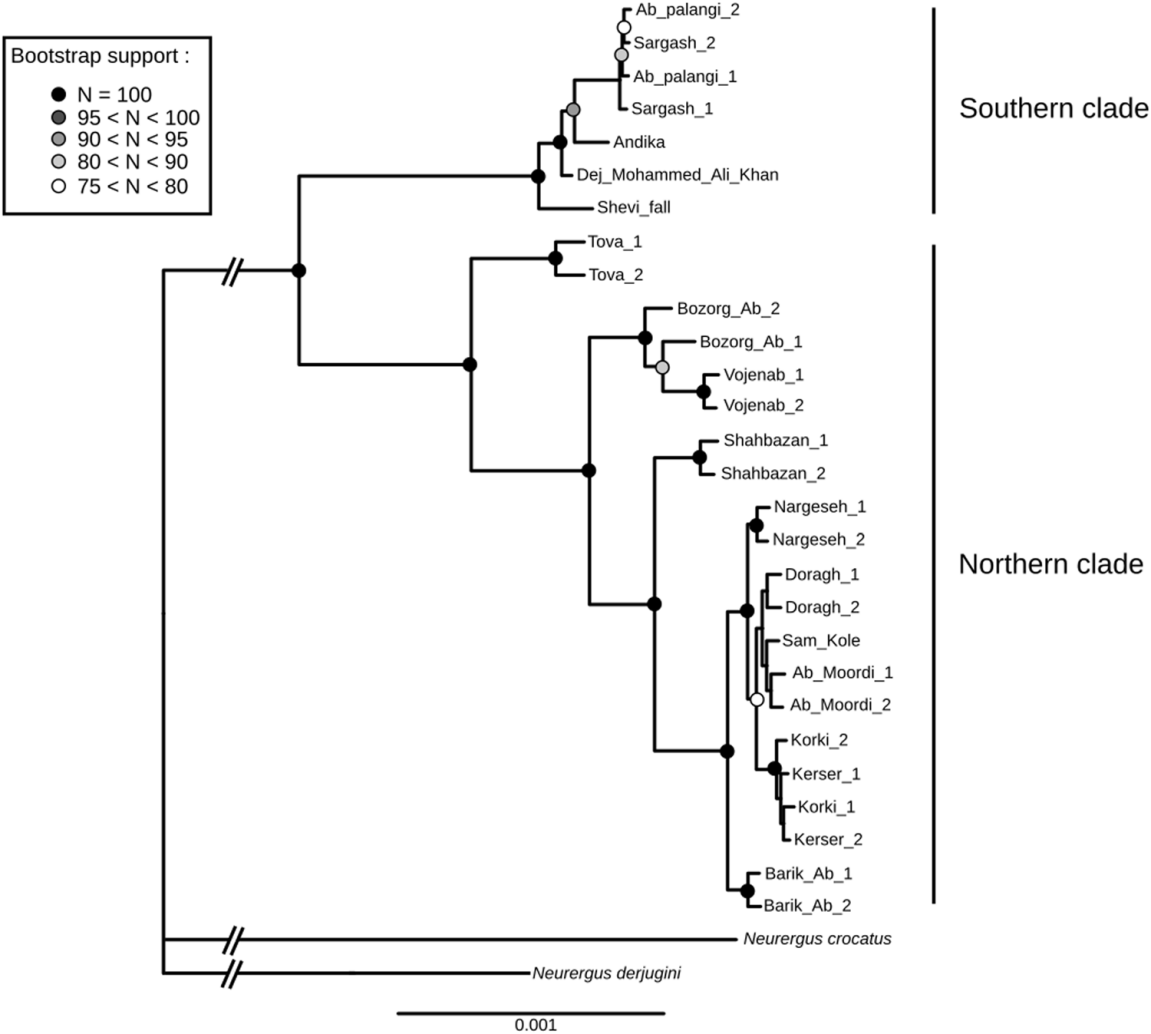
Phylogenetic tree of *Neuregus kaiseri* native to southwestern Zagros Mountains in Iran based on the concatenation of 23,518 RAD loci (2,709,284 bp), with *N*. *crocatus* and *N*. *derjugini* as outgroups.

Using a Maximum Likelihood approach on concatenated RAD loci (complete sequences) from the dataset with outgroups, we recovered a tree that is resolved for the majority of nodes (Fig. 2). This tree shows a structure within *N*. *kaiseri* that is consistent with the previous study on a segment of the mitochondrial DNA^24^, populations are split into two monophyletic groups, which are geographically separated by the Dez River (Fig. 1). While the Southern group is rather homogeneous, the Northern one displays strong genetic diversity, and can be further separated in 4 monophyletic groups of populations: Tova, Bozorg-Ab + Vojenab, Shahbazan and a larger group that ranges from Barik-Ab in the South to Kerser in the North (thereafter referred to as ‘core Northern populations’).

**Figure 3:**
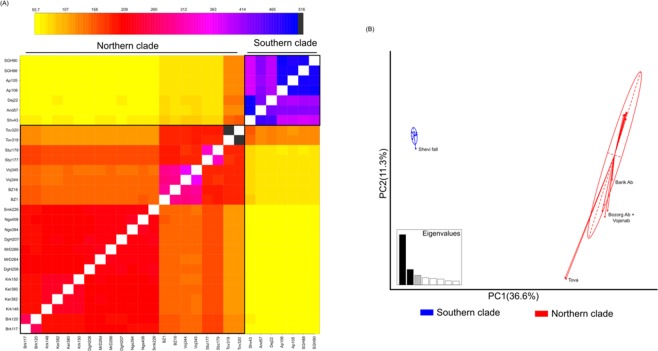
(**A**) Co-ancestry matrix of populations of *Neurergus kaiseri* native to southwestern Zagros Mountains in Iran inferred with fineRADstructure, represented as a heatmap. (**B**) Representation of the first two axes of the PCA, with individuals coloured depending on the clade they belong to. For each clade, an inertia ellipse containing 67% of the individuals is represented.

In line with the phylogenetic tree, a fineStructure analysis recovered two groups corresponding to the populations North and South of the Dez River (Fig. 3A). Higher co-ancestry levels were found for pairs of individuals from the same clade. While the co-ancestry among individuals from the Southern clade is very high, individuals from the Northern clade show hierarchical levels of genetic structure reflecting the lineages recovered on the phylogenetic tree. The levels of co-ancestry between individuals from the Southern and Northern clades gradually increase when getting closer to the Dez River, with the individuals from Tova sharing more ancestry with the Southern clade’s individuals than with those from the ‘core Northern populations’.

The Northern and Southern clades were also very distinct on the first axis of the PCA (accounting for 36.5% of the variance; Fig. 3B) with no intermediate individuals between them. Still, the populations of Tova, Bozorg-Ab, Vojenab and Barik-Ab are slightly closer to the Southern clade. On the other hand, both groups are not differentiated on the 2nd and 3rd axis (accounting for respectively 11.3% and 7.0% of the variance), which can be attributed to the intra-clade differentiation. The variation at these axes confirms that the Southern clade is very homogeneous, while the Northern clade shows a structure in line with the previous analyses.

### Factors affecting genetic differentiation

An AMOVA analysis of the complete dataset revealed that factors such as the sampling location and the clade were explaining significant amount of genetic variation among populations (*p* = 0.01 for both). The variation between clades accounted for 76.62%, while the variation between populations within the clades accounted for 16.25%. When performing the AMOVA only on individuals of the Southern clade, geographic location North or South of the Karoon River was significantly correlated with genetic differentiation (*p* = 0.01) explaining 45.24% of the total variance.

**Figure 4:**
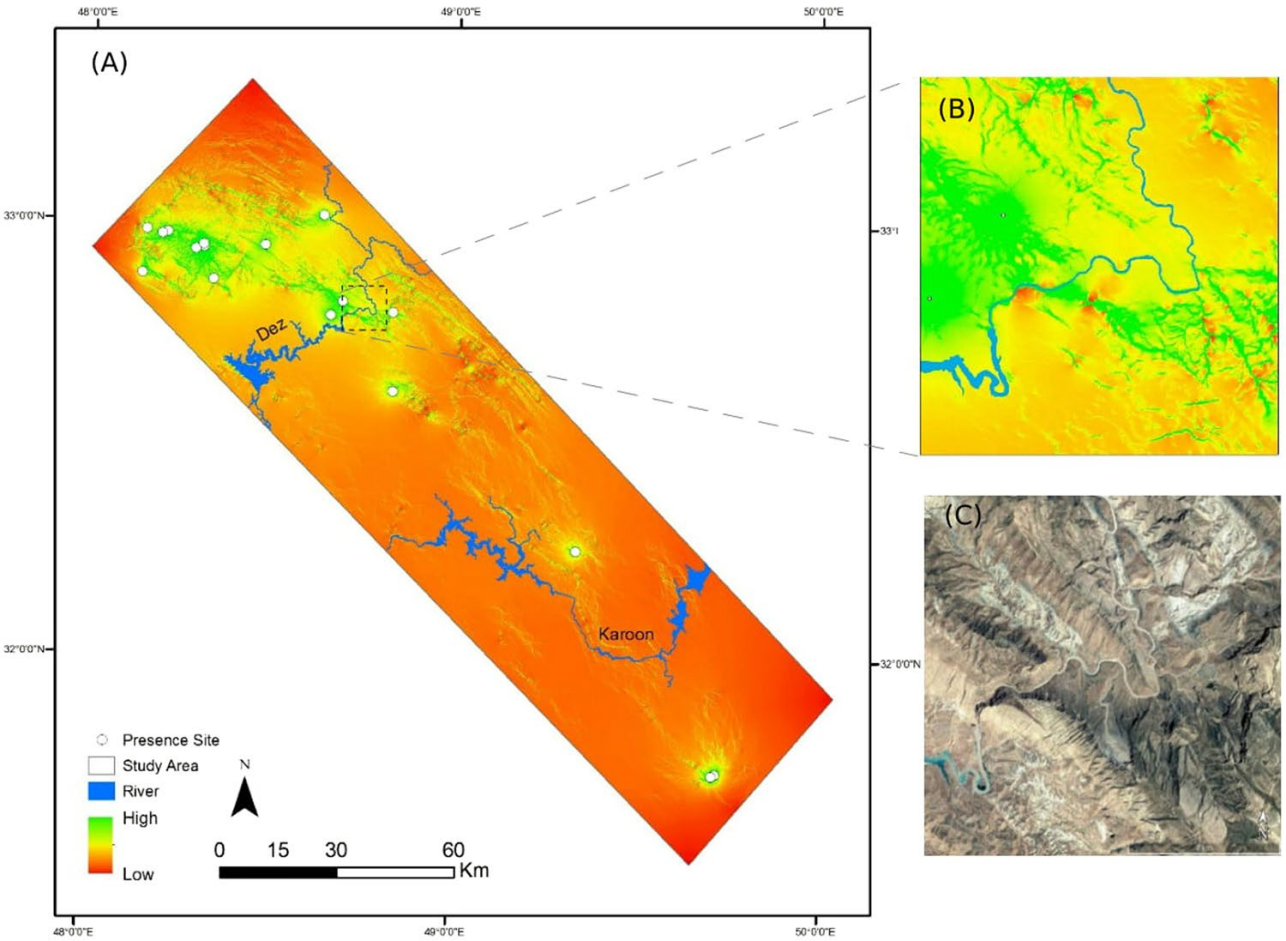
(**A**) Circuitscape cumulative current map of the density of potential movement of *Neurergus kaiseri* between 16 water bodies across southwestern Zagros Mountains, Iran. Petential movement corridors of the species are indicated by  currents flowing through the landscape ranges from high (green colour, indicating the high connectivity), to low (red colour, indicating the limited movement). (**B**) The enlarged section of fragmented distribution range by the Dez River and (**C**) the satellite imagery of that section obtained from Google Earth (Image ^©^2018 CNES/Airbus; Imagery date: 5/19/2017).

Mantel tests revealed a significant relationship between genetic distance and log-transformed geographic distance (isolation by distance) (Table 1). ‘Isolation by resistance’, and ‘isolation by river’ significantly influenced pairwise population genetic differentiation as genetic distance significantly increased with increasing pairwise landscape resistance. In ‘cumulative map of currents’, high-flow areas represent possible pathway of dispersal (Fig. 4A–C). Mantel test detected a signal of isolation by environment as climate dissimilarity had significant influence on genetic distance. Each of four variables in isolation had a significant effect on genetic differentiation and then included in the multivariate model.

**Table 1:** Mantel test, and variable importance and significance (*p*) of Generalised Dissimilarity Modelling (GDM) to assess the association between genetic distance and geographic distance (isolation by distance), landscape resistance (isolation by resistance), river disconnectivity (isolation by river), and environmental dissimilarity (isolation by environment) in *Neurergus kaiseri* populations across southwestern Zagros Mountains, Iran.

Variables	Source	Mantel *r* (*p*)	Importance in GDM (*p*)
Geographic distance (IBD)	Geographical position	0.67 (0.00)	8.38 (0.00)
Landscape resistance (IBR)	Circuitscape output	0.59 (0.04)	0.00 (0.75)
River disconnectivity (IBRiv)	Circuitscape output	0.81 (0.00)	34.97 (0.00)
Environmental dissimilarity (IBE)	Bioclim data set	0.38 (0.00)	0.00 (0.73)

**Figure 5:**
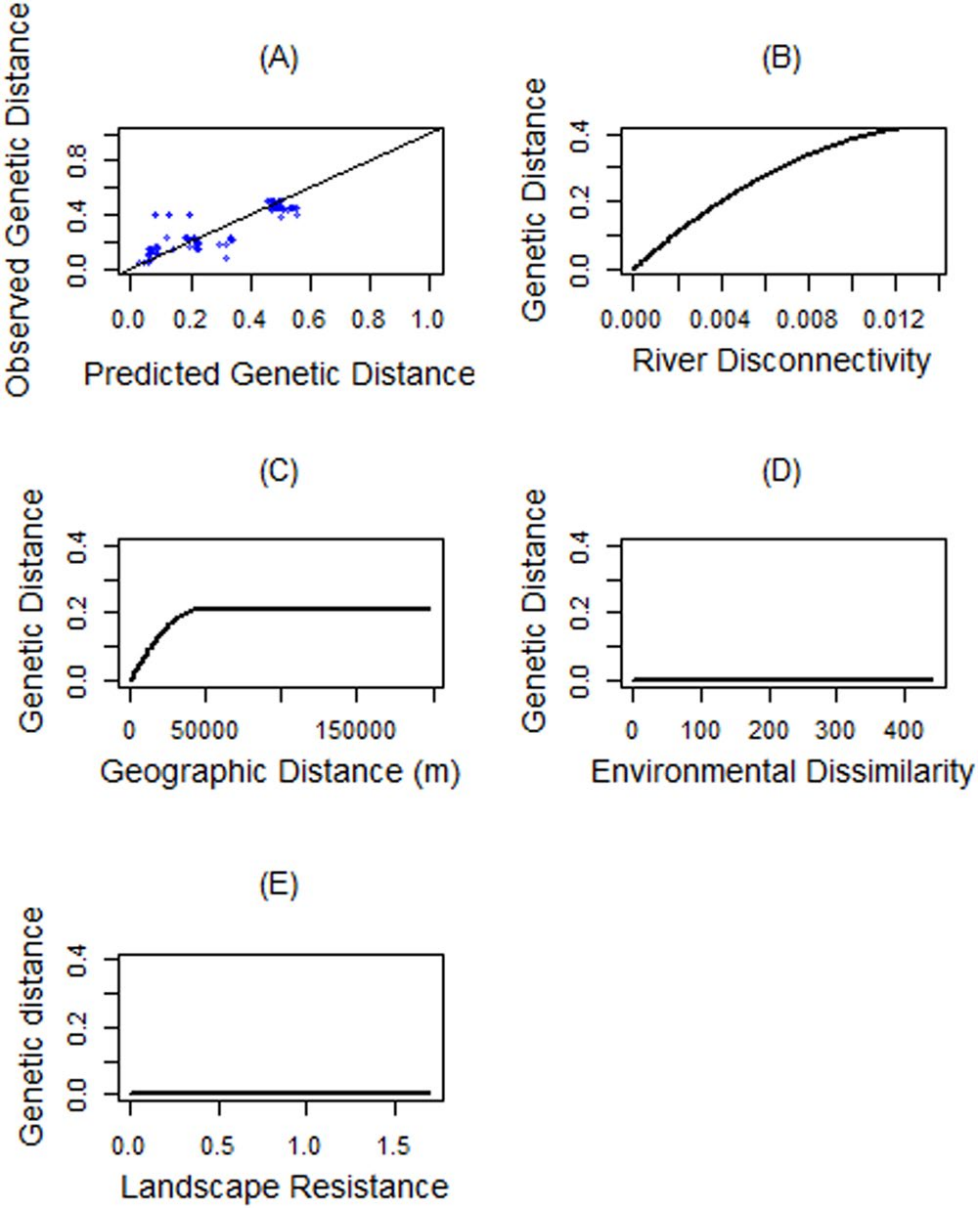
Generalised dissimilarity modelling (GDM) of variables associated with genetic differentiation in *Neurergus kaiseri*. (**A**) Relationship between predicted and observed pairwise genetic distance. Blue dots are site pairs, and the line represent a perfect fit for the function relating observed to predicted genetic differentiation. (**B**) Predicted spline showing the estimated non-linear relationship between genomic distance and river disconnectivity. (**C**) The geographic spline showing increasing genomic variation as geographic distance increases up to 50 km and no predicted genomic change between sites far than 50 km apart. The genomic distance does not change by increasing environmental dissimilarity (**D**) nor landscape resistance (**E**).

To test correlations between site-by-site genetic distance matrix and its predictive factors, we used Generalised Dissimilarity Modelling (GDM). The fitted GDM explained 74% of genetic differentiation between populations. Visual examination of the genetic distances predicted from the model versus the observed values indicated that the model had reasonable predictive power (Fig. 5A). The GDM showed a positive strong non-linear relationship between genetic distance and river disconnectivity (Fig. 5B), meaning rivers explain 66% of genetic deviance and the remaining 8% deviance is caused by geographic distance (Table 1). As outlined by Fig. 5C, genetic differentiation increases sharply with geographic distance up to 50 km and remains stable beyond. In contrast, environmental dissimilarity and landscape resistance had no important effects on observed genetic variation (Fig. 5D,E and Table 1).

### Phylogenetic niche conservatism

**Figure 6:**
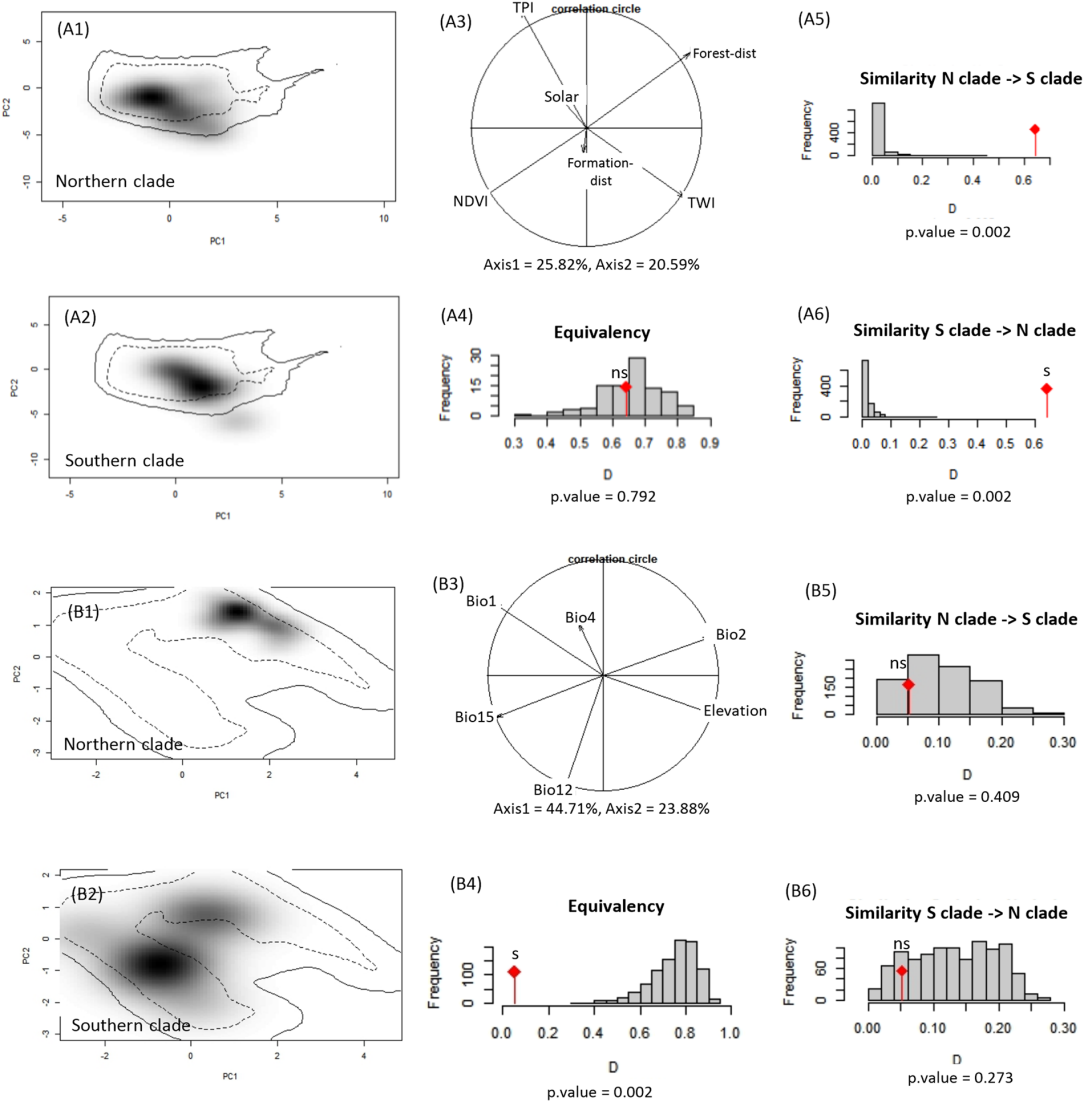
Niche of the two clades of *Neurergus kaiseri* at local (A) and landscape (B) spatial scale across southwestern Zagros Mountains, Iran. Black shading indicates the density of the occurrences of the Northern (**A1**,**B1**) and Southern clades (**A2**,**B2**) by cell. The solid and dashed lines, respectively demonstrate 100% and 50% of the delimited background climate. Relative contribution of each variable is shown in a PCA-env barplot (**A3**,**B3**). Histograms **A4** and **B4** compare the observed Schoener’s *D* with estimated *D* for randomly selected points at climatic space of the two clades. Histograms (**A5-6** and **B5-6**) show the observed niche overlap *D* between the two clades (bars with a diamond) and simulated niche overlaps (grey bars) on which tests of niche similarity are calculated. The significance of the tests is shown; ns: non-significant, s: significant (*p* < 0.05).

At the local habitat scale, PCA-env indicated that 46% of the environmental variation was explained by the first two PCs. There was no significant difference between occupied niches of the two genetically differentiated clades at this scale. The occupied climatic niches by the two clades were statistically equivalent (*p* = 0.79) and similarity of them was significantly more than expected by chance (*p* = 0.02). Accordingly, niche conservatism hypothesis could not be rejected at the local scale (Fig. 6A, 1–6).

The first two components of PCA-env explained 68.59% of the total variation along climatic gradients (PC1 = 44.71%, PC2 = 23.88%). The Northern clade is rather restricted at both dimensions of the climatic space, while the Southern clade covers a wide climatic niche along both PC1 and PC2 (Fig. 6B, 1–3). In agreement with the relatively low value of the Schoener’s *D* metric (*D* = 0.04), equivalency test illustrated that the two clades occupied statistically non-equivalent climatic spaces (*p* value = 0.002) (Fig. 6B, 4). The observed low niche similarity of the Northern clade compared to the Southern clade is significantly not different by chance and vice versa (Fig. 6B, 5,6). PCA-env equivalency/similarity tests revealed that the occupied climatic niches by the two clades were not identical but had some similarities. It seems that the ancestral niche centroid of *N*. *kaiseri* has shifted towards areas that are warmer (1 °C on average) and receive more precipitation. The random translocation and rotation (RTR) test showed no significant signal of niche conservatism nor divergence at climatic space since the observed Multidimensional niche overlap (MO) was in the 95% of the null distribution (MO = 0.18; *p = *0.73) (Supplementary Fig. S1).

## Discussion

By reaching the Southern extent of the geographic range of salamandrids in Eurasia, Kaiser’s newt (*Neurergus kaiseri*) takes an outstanding position. Populations of *N*. *kaiseri* cover roughly an area of only 10000 km^2^ and are potentially threatened by local extinction^23^. Therefore, the detailed analysis of population structure is a crucial basis for the effective conservation of this species in the future. Our detailed ecological niche modelling and landscape genetics approach allows for the identification of migration paths and parameterisation of the landscape factors affecting functional connectivity between sites inhabited by Kaiser’s newts. The results from our study are essential for landscape planning in order to avoid loss of genetic diversity due to inbreeding and genetic drift^26,27^ caused by habitat fragmentation.

With our extensive dataset we could corroborate the presence of two major clades as indicated by a previous study based on mitochondrial markers^24^. These two clades are geographically separated by the Dez River resulting in a ‘Northern clade’ and ‘Southern clade’. Based on different genetic analyses both clades appear to be well separated, even in the populations bordering the Dez River (the closest populations of the two clades being only 13 km apart). We interpret these results as a sign for a reduction, or even absence, of gene flow between the clades. Most likley, the Dez River has been acting as a barrier to gene flow. However, the co-ancestry between individuals from the Northern and Southern clades gradually increases when getting closer to the Dez River, as expected under a scenario of isolation by distance. The individuals from Tova (Northern clade), for example, show an intermediate state of co-ancestry, being more close to the Southern clade than to the Northern-most populations. Thus, it seems that the presence of a barrier to migration alone can’t explain the observed pattern of genetic variation.

The present analyses also revealed some interesting patterns of genetic diversity within the two clades. Even though populations of the Southern clade are separated by relatively large geographic distances, up to 145 km, they differ at ‘only’ 20% of the SNPs, while in the Northern clade the maximal observed genetic divergence is of 34% for a maximum geographic distance of 53.5 km. Within the Northern clade, we can further delimit four groups: (i) a core group of populations that are rather homogeneous and formed of most of the northernmost localities; (ii) the population of Shahbazan, which is still closely related to the core group although geographically distant; (iii) the populations of Bozorg Ab and Vojenab, and iv) finally the population of Tova. One possible explanation for these different patterns of genetic diversity within clades might be that during Quaternary climatic oscillations, the Northern part of the Zagros mountains acted as a refugium for Kaiser’s newt, while the Southern part was colonised more recently as climatic conditions became more suitable. Similar climatic processes have been assumed for shaping diversity of brown frogs in the Near East. Here, climatic oscillations triggered post-Messinian speciation events and populations could only survived in humid refugial areas during the glacial periods in the Northern Hemisphere that resulted in cold and dry weather conditions in that area, while an expansion was only possible during the warmer and more humid interglacial periods^28^. In *N*. *kaiseri*, a southward colonization/expansion from a northern refugium, followed by the spilt of the populations in two groups due to the opening of the Dez River would be a reasonable scenario in this context. However, studies on the effect of past climatic oscillation on population structure and patterns of genetic diversity of biota in the Zagros Mountains are missing and therefore it is highly speculative to link patterns of observed genetic diversity with specific geological as well as climatic events.

Interestingly, the divergence within and between clades on the PCA are not correlated to the same axes (see Fig. 3B), suggesting that these processes are linked to distinct loci in the genome. One possible explanation for this outcome could be that the divergence between clades has been mainly driven by genetic drift, promoted by barriers to migration or distance gradients, while divergence within clades could be linked to other processes, such as local adaptation that would involve different loci. Similar findings could be revealed from the well-studied salamander system – the Fire salamander (*Salamandra salamandra*) in Europe – where phylogenetic species divergence and local adaptation are realised by different sets of genes (Goedbloed *et al*. 2017; Czypionka *et al*. 2018). Unravelling the general processes as well as genetic mechanisms associated with population structure of *N*. *kaiseri* at different levels will be therefore rather interesting from a general evolutionary perspective.

By analysing various habitat and ecological parameters of populations of *N*. *kaiseri*, we were able to quantify the role of ecological and geographical forces, such as IBD, IBR, IBRiv, and IBE in shaping genome-wide variation of Kaiser’s newt. As a result, we found that all four factors in isolation had an impact on genetic differentiation among *N*. *kaiseri*’s populations. In line with recent findings, we infer that the spatial arrangement of populations across landscape is determined by IBD, IBR, IBRiv, and IBE and can contribute to spatial genetic divergence^29–31^. In our study, IBRiv and IBD explained 74% of the genetic variation between populations. Some variation in genetic distances of the populations could be attributed to genetic drift as a consequence of intensive harvesting that occurred at distinct times at several places.  Although mtDNA-based analysis showed that IBD is the main driver of genetic differentiation of *N*. *kaiseri* populations^1^, genome-wide sampled single nucleotide polymorphisms (SNP data), which are expected to be more informative and sensitive, indicated that rivers can be the main factor for disconnectivity of populations causing genetic divergence. Geographic distances between *N*. *kaiseri* populations ranged from only 0.669 km up to 201 km. We showed that up to a scale of 50 km, geographic distance showed a measurable effect on genetic variation (differentiation at 20% of the SNPs). Beyond this distance, migration of newts seems to be not possible with gene flow being completely disrupted. Such a high distance is completely unexpected for a newt species at the southern edge of the Salamandridae’s distribution and fits nicely to the changing view that salamanders and newts do have a high dispersal propensity (see^16,32,33^).

The main clades of *N*. *kaiseri* follow a parapatric distribution pattern with displaying strong genetic differentiation between clades, suggesting that a physical barrier exists to prevent gene flow between populations. The apparent disjunction of the two clades is probably due to the high physical landscape heterogeneity, resulting in a hard allopatry^14^. Landscape barriers can influence genetic structure of populations and can lead to inbreeding or loss of genetic diversity^34^. The cumulative current map illustrates two key water resources, Karoon and Dez Rivers, which may act as physical barriers to migration and gene flow of newts. Since the Dez River separates populations of the Northern and Southern clades, ‘isolation by river’ hypothesis is strengthened under  this scenario. As confirmed by AMOVA analysis, there is a significant genetic differentiation between the two clades located on opposite sides of the Dez River. The physicochemical characteristics of a river can impact migration of amphibians. With an estimated discharge of 245 m^3^ s^−1^ an annual suspended sediment load of 7.5 million tonnes (see Fig. 3C)^35^ this river might have the potential to prevent migration of newts. Lemmon, *et al*.^36^ demonstrated that the Teays-Mahomet River acted as a barrier between *Pseudacris ferarium* and *P*. *triseriata*, resulting in allopatric speciation. In contrast, for newts of *Triturus* estimated intra-specific gene flow across the Tejo River was higher than expected^37^. Still, rivers in general seem not to build a strict barrier to migration of Kaiser’s newts. Karoon River had a medium effect on genetic differentiation within the Southern clade, as here the results of the AMOVA analysis  indicated less than 50% genetic difference between populations on the both side of this river. The river bed of Karoon has been extended due to various dams constructed across the river over the half past century. Accordingly, newts may have previously passed through the Karoon to occupy new springs on the other side, but there is no historical evidence to prove that.

We assessed the niche of these two clades. The centroid climatic niche of the Southern clade was far beyond the climatic tolerance of the Northern clade. Accordingly, populations of the different clades may occupy different realised niches within their ancestral fundamental niche^14^. The random similarity of the climatic niches of the two clades beside a strong non-equivalency between them supports the hypothesis of niche constraint with a southward shift in the Southern clade to a warmer area with higher annual precipitation and lower elevation. Evidence of a southward niche shift has been reported during the last glacial period for different amphibians in Europe; members of the genera *Rana* and *Triturus* expanded southwards and re-extended ranges across northern and central Europe post-glaciation^38^. However, exhibiting equivalent and similar niches at local habitat scale implies that the niche of *N*. *kaiseri* has been conserved.

We showed that the niches of the two clades tend to be conserved in geographically separate ranges. GDM result supportively rejected the *a priori* hypothesis that there is a ‘barrier of adaptation’ (i.e. IBE) for the successful establishment of dispersers between sites. Therefore, our data suggest that local adaptation probably plays a less important role in *N*. *kaiseri* and that genetic differentiation among populations is mainly the result of other underlying evolutionary processes.

After the divergence of *N*. *kaiseri* from *N*. *crocatus* approximately 5 million years ago (mya) in the late Miocene^20^, Kaiser’s newt dispersed across the Zagros Mountains’ front detrital apron. The origin of stream networks in this area, including the Dez River, is suggested to have occurred ~3–3.5 mya in the late Pliocene^39^. *N*. *kaiseri* then extended its distribution range southwards and migrants could occupy suitable terrestrial and aquatic habitats for their reproduction. Consequently, it can be assumed that the two major clades of *N*. *kaiseri* diverged about 1–1.2 mya in the middle Pleistocene^24^. Overall, high physical and ecological variation may have promoted divergence of the two clades in presence of PNC. Since there were no transition zones between the ranges of the two clades, the evolution of reproductive barriers and gradual speciation seems to be likely.

Consistent with PCA-env, results of the RTR analysis did not support niche conservatism or divergence in climatic space, strengthening the niche constraint pattern. At a local habitat scale, the two clades occupied the closest analogue of the ancestral niche, but in different climatic conditions, while expanding their ranges on opposite sides of Dez River. Niche similarity and equivalency tests suggested ‘niche constraint’ pattern of PNC by showing the differences in climatic niches between the two clades and a southward shift in niche centroid over time. Ecological specialisation in Kaiser’s newt, as in other amphibians, depends on different processes that occur at multiple spatial scales. As the species occupy similar habitats at the local-scale in alternate climatic conditions, adaptive (ecological) divergence of populations is more probable when niche shift occurs at the local habitat space.

The high genetic differentiation – both on the mitochondrial as well as on the nuclear genome-wide level - between the Northern and Southern clades of *N*. *kaiseri* raises the question of whether these two lineages should be considered as distinct species. In a recent study analysing phylogenetic relations and investigating species boundaries of species of *Neurergus*, Multi-Species Coalescent (MSC) models consistently split the Southern and Northern clades of *N*. *kaiseri* in two species with high support^22^. However, MSC models tend to ‘over-split’ lineages^40^, and it is then important to test species hypotheses with other approaches. At a first glance, both clades appear to be really separated based on genome-wide differentiation of SNPs, no signs of recent hybridisation or ongoing gene flow (see Fig. 3B). However, the closer inspection of pairwise co-ancestry between the sampled individuals indicates that when getting closer to the ‘contact zone’, Northern populations have a higher relatedness with the Southern ones. Thus, it seems that at least one migration event has occurred from the Southern to the Northern clade, resulting in admixture. Analysis of the different factors correlated with genetic differentiation suggested that, while IBRiv is the main factor, IBD plays an important role as well. It is thus possible that, due to the low dispersal abilities of this newts and the presence of strong barriers to gene flow, a strong genetic structure rapidly emerged within the group, without reflecting reproductive isolation that could grant species status to the two clades. Furthermore, the observed niche conservatism between both clades further indicates a lack of ecological divergence.

## Methods

### Sampling and genetic analysis

A field survey was conducted across the entire *N*. *kaiseri* distribution range in southwestern Iran in 2015. Out of 30 breeding ponds recorded for this species, we choose to sample only 16 sites for our study (Fig. 1). The ponds not included were close by to one the sampled sites (less than 500 m apart) and located in homogenous landscape and similar climatic conditions. We took tissue samples from tail tips (~1 cm) of larvae without scarifying them for later extraction of genomic DNA as suggested by Polich, *et al*.^41^. The Iranian Department of Environment issued permission for sample collection.

For the generation and assembly of the RADseq dataset we took 1 or 2 individuals per population, resulting in a total number of 28 individuals representing 16 populations/locations of *N*. *kaiseri*. One individual each of *N*. *crocatus* and *N*. *derjugini*, representing two closely related species, were used as outgroups for phylogenetic analyses. Genomic DNA was extracted using the Macherey-Nagel NucleoSpin Tissue kit following the manufacturer’s instructions. We performed double-digest Restriction Site Associated DNA sequencing (ddRADseq^42^) preparing the library as follows^25^: 1 μg of DNA from each individual was double-digested using the PstI-HF and AclI restriction enzymes (NewEngland Biolabs) and modified Illumina adaptors with unique barcodes for each individual were ligated to obtained DNA fragments. Samples were multiplexed (pooled) and a Pippin Prep was used to select for fragments with a size around a tight range of 383 bp, based on the fragment length distribution identified using a 2200 TapeStation instrument (Agilent Technologies). Finally, enrichment PCR was performed to amplify the library using forward and reverse RAD primers. Sequencing was conducted on an Illumina Next-Seq machine at Glasgow Polyomics to generate paired-end reads 75 bp in length.

De-multiplexed reads were assembled *de novo* using ipyrad^43,44^ and clustered using a 95% similarity threshold; base positions with a coverage >8 were called. Finally, since uneven missing data among populations can have deleterious impact on downstream analyses, loci with less than 28 individuals covered were discarded from further analysis. The assembly was performed with two different subsets: one containing all the 30 samples, and one with only the 28 samples of *N*. *kaiseri*. Indeed, as the number of RAD loci recovered is expected to decrease when the evolutionary distance between samples increases, the approach allows to maximise the amount of loci used in analyses that do not necessitate the outgroup taxa.

### Genetic structure of the populations

We used the concatenation of the loci from the dataset with outgroups to infer a phylogenetic tree with a maximum likelihood approach. Accordingly, we used the hill-climbing algorithm implemented in RAxML^45^, with a GTR + GAMMA substation model, and performed 100 bootstrap replicates.

As phylogenetic models can give an incomplete or even misleading representation of the populations history^46^, we also investigated the genetic structure of *N*. *kaiseri* using various population genetics approaches. First, we used SNPs extracted exclusively from the kaiseri populations dataset to estimate the co-ancestry between each pair of individuals using fineRADStructure^47^. Then, in order to understand the relationships between the genetic groups and detect potential hybrids, we used the SNPs as variables to perform Principal Component Analyses using the ‘adegent’ R package^48^. More precisely, we represented the individuals in a multivariate space depending on their centred-scaled allele frequency at each SNP. We then defined Principal Components (i.e. axes maximizing the variation of the variables in the multivariate space) in order to reduce the number of dimensions, and studied the repartition of the individuals in the space defined by the PCs.

### Factors affecting genetic differentiation

In order to analyse whether observed genetic variation of individuals is distributed among or within clades, we performed an Analysis of Molecular Variance (AMOVA^49^) on the 18,546 SNPs, using the ‘poppr’ package^50^ in R. The statistical significance of the results was assessed based on 99 permutations. A similar analysis was performed on a subset including only the individuals from the Southern clade, to test for the effect of the Karoon River on genetic differentiation. The individuals were separated in two groups depending whether they were located between the Dez and Karoon Rivers (populations Shevi fall, Dej Mohammad-Ali Khan, Andika) or South of the Karoon River (populations Ab-Palangi, Sargach).

We estimated the pairwise genetic differentiation between localities using the SNPs from the RADseq loci. However, since the sampling size for each site is very low (1 or 2 individuals), widely used estimators such as *F*_*ST*_ can be inaccurate^51^. Thus, we calculated the genetic distance between individuals (i.e. the percentage of SNPs at which 2 individuals differed), and then averaged them to get the pairwise distance between populations. We recorded geographic position of presence sites over the sampling period. Pairwise Euclidean distances corresponding to straight-line geographic distances between presence sites were estimated using the ‘vegan’ R package^52^. We used log-transformed geographic distance to rescale predictor variables in the same range.

To obtain a more accurate suitability map, we used 12 additional presence points along with the geographic locations of the 16 sampled populations. We created landscape resistance using all 28 trimmed presence points and six environmental predictor variables. All information layers including the normalised difference vegetation index (NDVI), solar radiation, Topographic Position Index (TPI), Topographic Wetness Index (TWI), distance to the conglomerate formation and distance to the forest were mapped at 30 m spatial resolution in ArcGIS 10.4. NDVI quantifies vegetation density by measuring the difference between near-infrared, which is strongly reflected by the vegetation, and red light, which is absorbed by the vegetation. We used non-cloudy images of Landsat-7 ETM to measure NDVI values. Solar radiation is the power per unit area received from the sun and effects on species habitat suitability by controlling biological process^53^. We created an average solar radiation map for the 1st and 15th of each month, hourly, from March to October (active season of the target species) using the Solar Radiation tool. TPI represents topographic roughness by comparing the elevation of each cell in a digital elevation model (DEM) to the mean elevation of neighbourhood cells. We prepared TPI layer using Land Facet Corridor Tool^54^. TWI quantifies effects of topography on hydrological processes and refers to the soil moisture content of each cell in the landscape. To consider geological effects on the possible migration of newts we calculated the Euclidian distance to conglomerate formations in the study area (i.e. Kashakan and Bakhtiari formations). Due to their high porosity these formations store water underground and might act as temporal habitat for transient newt individuals. As Kaiser’s newt is highly dependent on the forest which can provides heat shelter for migrants, we also calculated Euclidian distance to forest land cover. We included both distance layers in the modelling process (all maps are available at Supplementary Fig. S2).

We presumed that the low mobile species passes from pixels at the landscape that meets multidimensional requirements of its Grinnelian niche (corridor dwellers^55^). First, for each clade we predicted the suitability value of each pixel of the study area through Maxent modelling. We then used the ‘maximum’ overlay method by overlaying the two suitability maps and giving the output cells the maximum value of the two overlapping cells. This approach permits one-way dispersal of a clade. We then quantified the resistance layer (R) as opposite of the suitability (R = 1 − S). Finally, we calculated connectivity between populations in Circuitscape 4.0.5^5^. By assuming that individuals’ movement is limited across an area with high resistance (i.e. unsuitable area), Circuitscape estimates the density of potential dispersers through a landscape. This program revealed potential paths with the expected high density of dispersers. We used circuit distances matrix, calculated by Circuitscape, as resistance-based geographic distance. On the river raster, following Oliveira, *et al*.^56^, we assigned a value of 1 to main rivers in the study area as potential barrier of newt migration, and used Circuitscape to calculate the pairwise connectivity of populations that may be disconnected by the course of rivers.

We created and mapped 19 bioclim layers using ‘dismo’ R package^57^ based on climatology data of 20 synoptic stations around the study region. Besides elevation, we selected five bioclim variables including annual mean temperature (Bio1), mean temperature diurnal range (Bio2), temperature seasonality (Bio4), annual precipitation (Bio12), and precipitation seasonality (Bio15) based on physiological requirements of the species. The pairwise correlation between these five climatic variables was ≤0.72. Values of bioclim data were then extracted at each presence point to create an environmental dissimilarity matrix using ‘ecodist’ R package^58^.

We considered geographic distance, landscape resistance, river disconnectivity, and environmental dissimilarity at presence sites as the affecting variables on genetic variation. The hypothesis of isolation of populations by each distance matrix was separately assessed using Mantel tests in R. Significant variables were then accounted for Generalised Dissimilarity Modelling (GDM) as a matrix regression to estimate the non-linear relationship between the genomic distance of populations and its drivers^59,60^.

### Phylogenetic niche conservatism

We used 28 trimmed records of *N*. *kaiseri* that belonged to identified clades in the previous step to examine PNC in Kaiser’s newt at landscape and local scales following Willis and Whittaker^61^. The local habitat variables were NDVI, solar radiation, TPI, TWI, distance to the conglomerate formation and distance to forest, as briefly described above. Species occurrences were then projected onto the gridded environmental space of the first two PCs calculated with the local habitat variables^62^. In addition, the five bioclim variables (e.g. Bio1, Bio2, Bio4, Bio12 and Bio15) were used to calculate the climatic niche similarity of the two clades at the landscape-scale. For this purpose, we applied PCA-env approach^62^ which characterised the climatic space at the two first principal components of the bioclimatic values for the entire study area per pixel. Thereafter, the probability of ecological niche evolution between these two clades was assessed based on the random translocation and rotation (RTR) approach following Nunes and Pearson^63^. RTR is a recently developed approach based on the Multidimensional Overlap (MO) metric to test the significance of niche differentiation. The MO metric is similar to BIOCLIM, a presence-only approach that takes into account species’ occurrences to delimit a climate envelope. It ranges between 0 (no niche overlap) and 1 (complete niche overlap). By maintaining the spatial configuration of occurrences, the RTR Null model calculates MO, niche overlap metric for thousands of replicates while randomly translocates and rotates the position of occurrences across the study region. The observed MO is finally compared to the distribution of MO values generated by the Null model. The RTR test also showed a great performance when dealing with rare, range-restricted species. Since RTR is suited for macro spatial scales and is highly dependent on relative occurrence area (ROA), we extended the study region to an extent in which 10,000 RTR replicates were possible without replacement.

## Supplementary information

Dataset 1

## Data Availability

The datasets generated and analysed during the current study are available from supplementary information and also the corresponding author on reasonable request. Assembly datasets used for the analysis are available at Mendeley data repository.
