# Supplementary material for: Geographic separation and genetic differentiation of populations are not coupled with niche differentiation in threatened Kaiser’s spotted newt (Neurergus kaiseri)
Source: Sci Rep. 2019 Apr 17;9:6239. doi: 10.1038/s41598-019-41886-8 (PMC6470216; doi:10.1038/s41598-019-41886-8)
Supplement: Supplementary file 1 — Dataset 1 [file 41598_2019_41886_MOESM1_ESM.docx]

**Geographic separation and genetic differentiation of populations are not coupled with niche differentiation in threatened Kaiser’s spotted newt (*Neurergus kaiseri*)**

Forough Goudarzi^1,2^, Mahmoud-Reza Hemami^1*^, Loïs Rancilhac^2^, Mansoureh Malekian^1^, Sima Fakheran^1^ , Kathryn R. Elmer^3^ and Sebastian Steinfartz^2^

*^1^ Department of Natural Resources, Isfahan university of Technology, Isfahan, 841568311, Iran*

*^2^ Department of Evolutionary Biology, Unit Molecular Ecology, Zoological Institute, Technische Universität Braunschweig, 38106 Braunschweig, Germany*

*^3^ Institute of Biodiversity, Animal Health & Comparative Medicine, College of Medical, Veterinary & Life Sciences, University of Glasgow, Glasgow G12 8QQ, UK*

*^4^ University of Leipzig, Institute of Biology, Molecular Evolution and Systematics of Animals, Talstrasse 33, 04103 Leipzig, Germany*

**Supporting Information**

**Appendix S1**


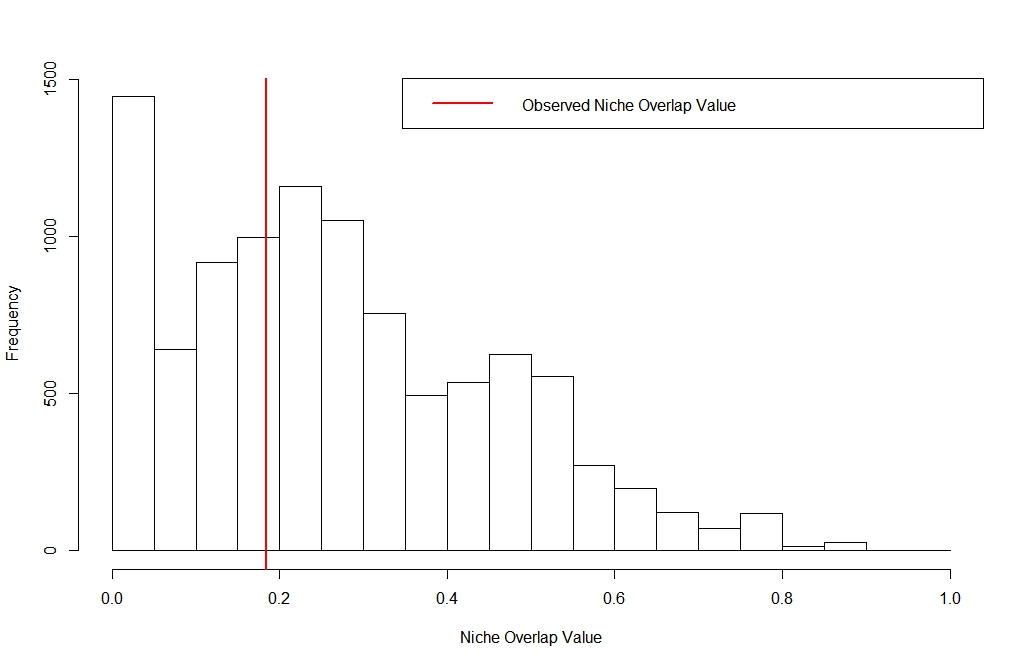


**Figure S1** The observed niche overlap of the northern and southern clades of *Neurergus kaiseri* in comparison against the null model based on MO metric (Multidimensional Overlap, Nunes and Pearson (2017)). The observed niche overlap (0.18) is in the 95% of the null distribution, so it supports neither niche conservatism nor niche divergence.

**Local-scale Environmental Variables**


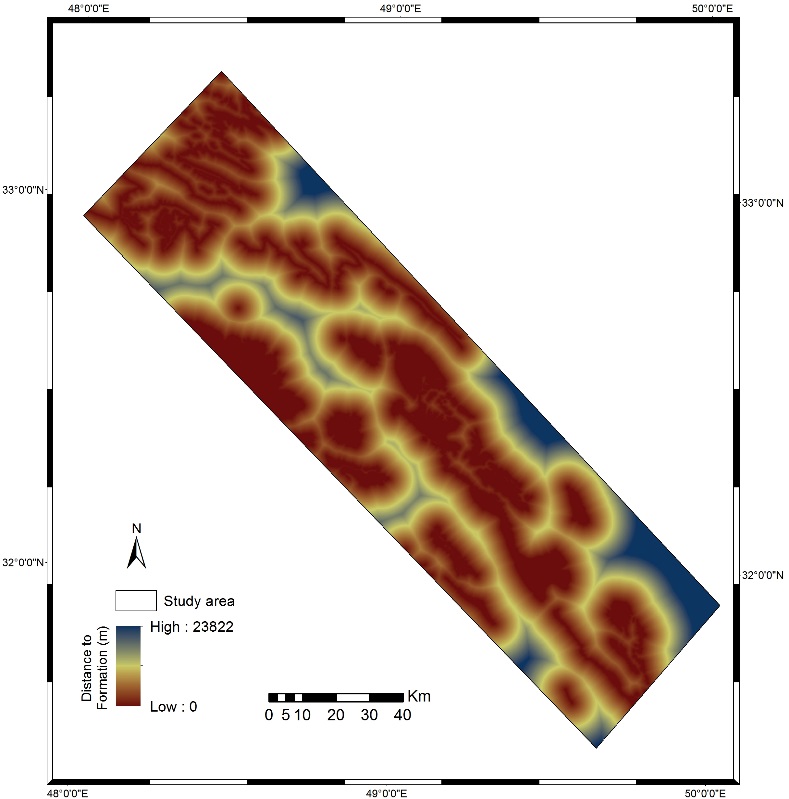

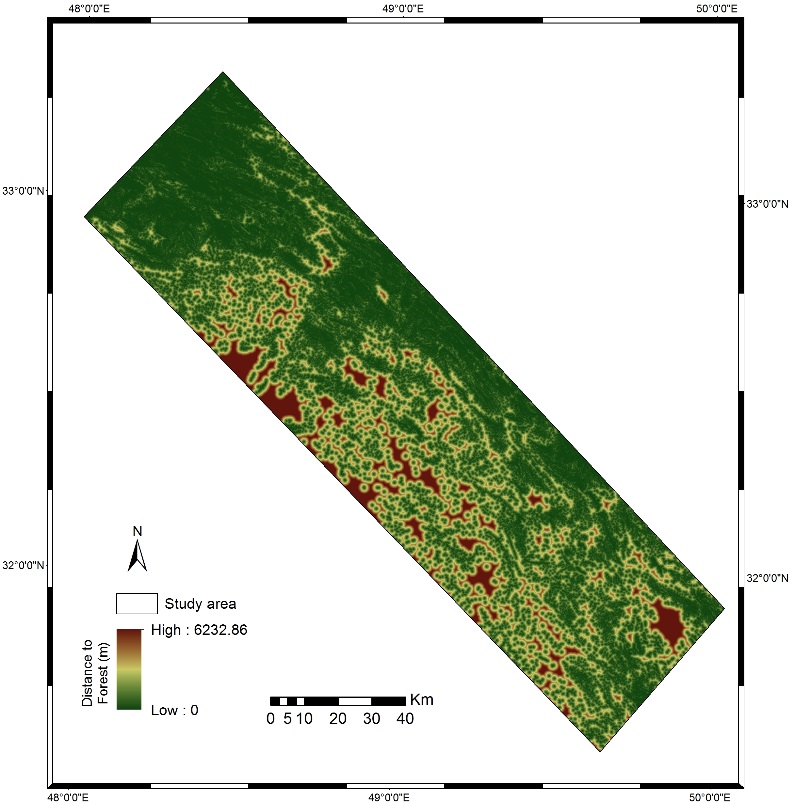

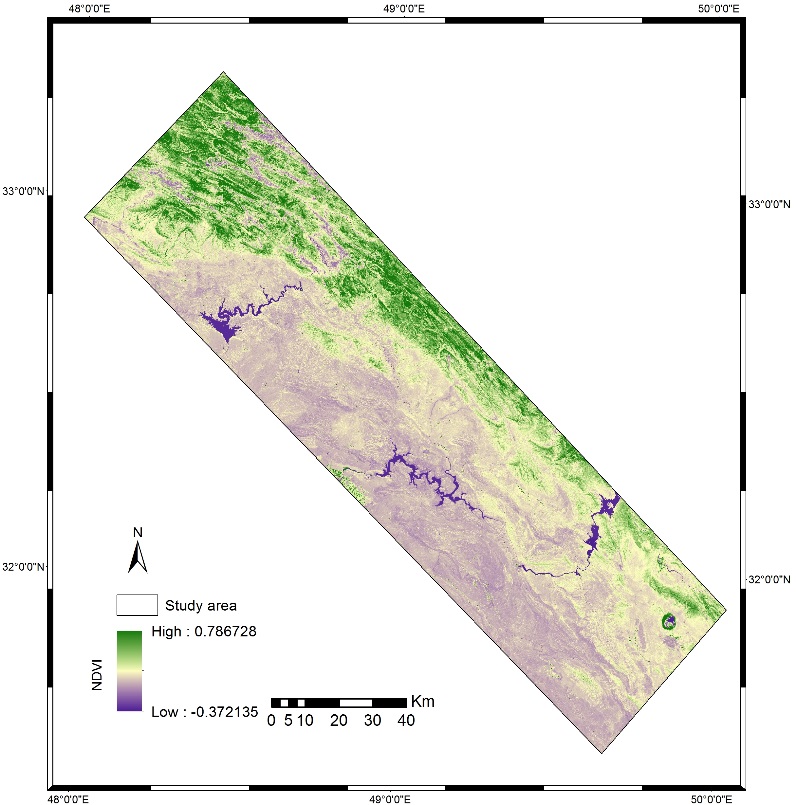

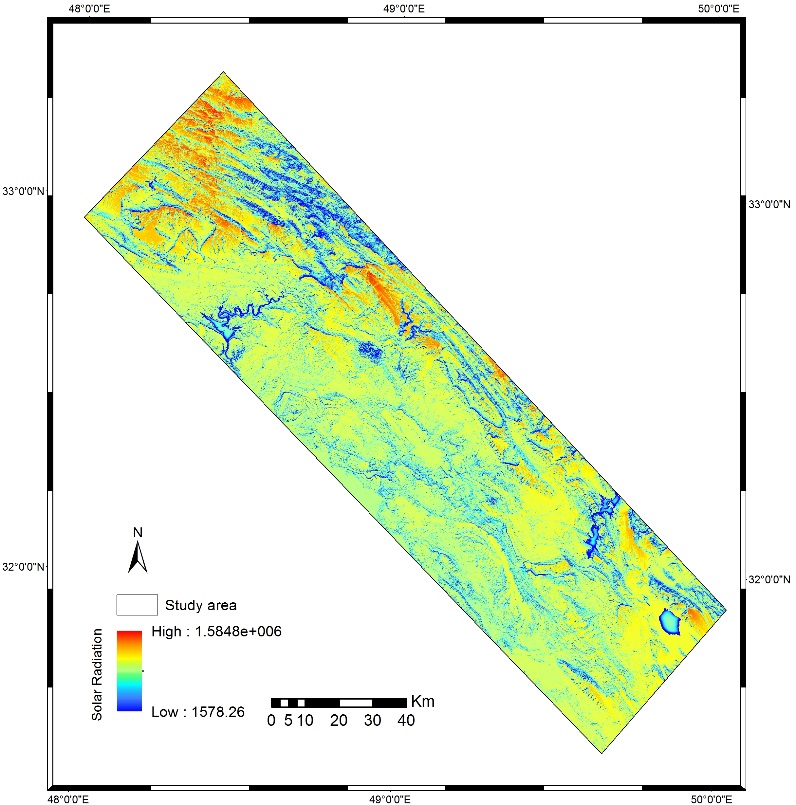


(A)

(B)

(C)

(D)


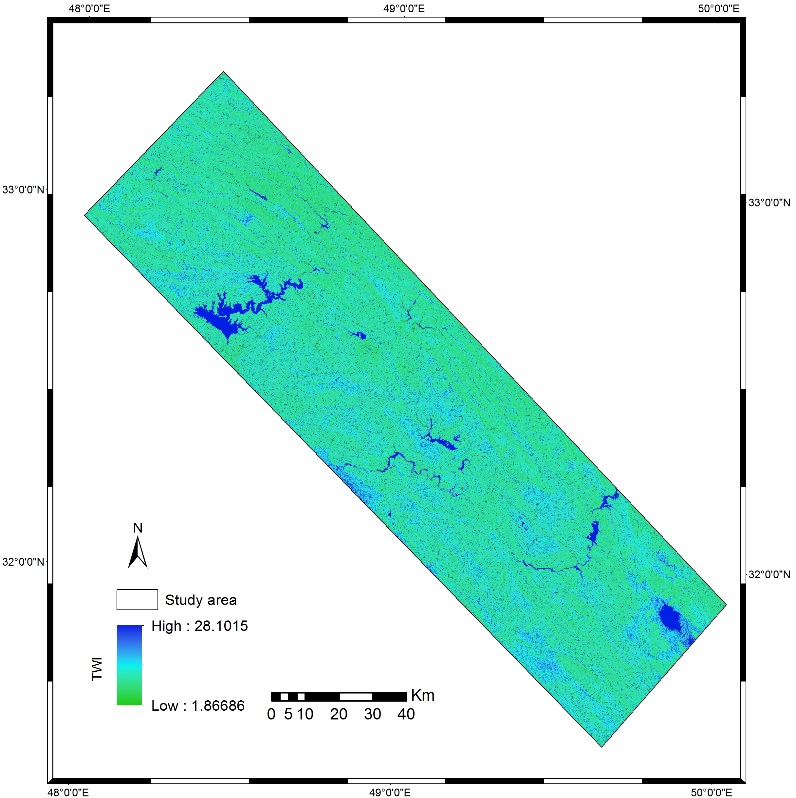

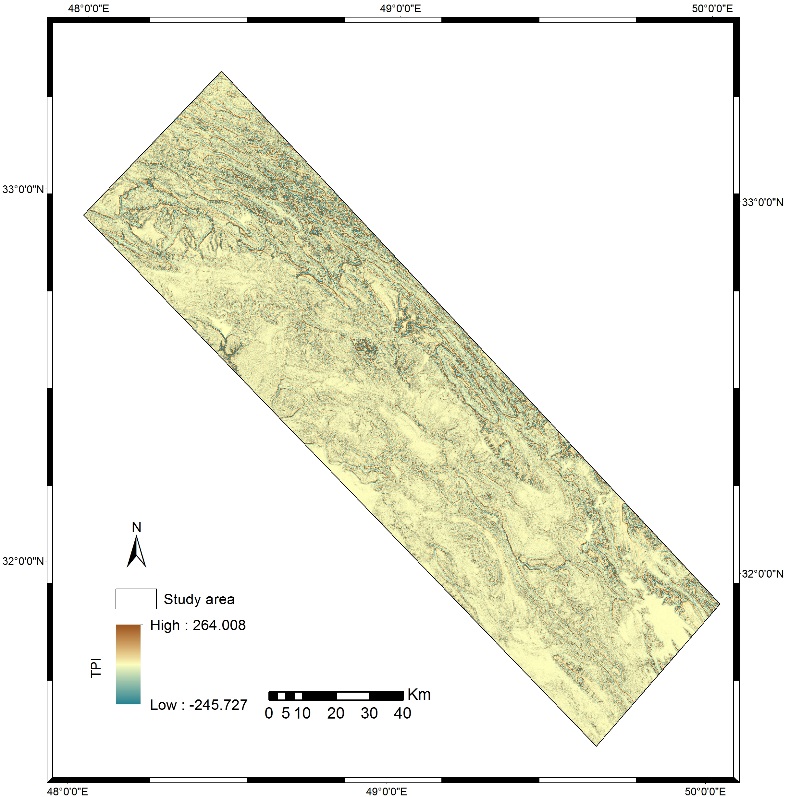


(E)

(F)

**Figure S2** The local-scale environmental variables including **(A)** the distance of each pixel in the landscape to the nearest forest polygon, **(B)** the distance of each pixel in the landscape to the nearest conglomerate formation, **(C)** map of [Normalised Difference Vegetation Index (NDVI), **(D)** map of solar radiation, **(E)** map of Topographic Position Index, and **(F)** map of Topographic Wetness Index. All maps were generated in ArcGIS 10.4.](https://en.wikipedia.org/wiki/Normalized_difference_vegetation_index)
